# Polyphenol estimated intake and dietary sources among older adults from Mallorca Island

**DOI:** 10.1371/journal.pone.0191573

**Published:** 2018-01-30

**Authors:** Joanne Karam, Maria del Mar Bibiloni, Josep A. Tur

**Affiliations:** 1 Research Group on Community Nutrition and Oxidative Stress, University of Balearic Islands & CIBEROBN (Physiopathology of Obesity and Nutrition CB12/03/30038), Palma de Mallorca, Spain; 2 Faculty of Health Sciences, University of Balamand, Balamand Al Kurah, Lebanon; 3 CIBEROBN (Physiopathology of Obesity and Nutrition CB12/03/30038), Madrid, Spain; Universitat de Lleida-IRBLLEIDA, SPAIN

## Abstract

The aim was the assessment of the polyphenol estimated intake and dietary sources among older adults from Mallorca Island. The study was carried out (2013–2014) in 211 participants dwelling women (*n* = 112) and men (*n* = 99). Polyphenol intake was calculated from two non-consecutive 24-h recall diets using the Polyphenol Explorer. The mean daily intake of polyphenol was 332.7 mg/d (SD: 237.9; median: 299 mg/d). Highest polyphenol intake was observed among females, 64–67 y.o. people, higher income and educational level, alcohol consumers, and physically active people. Most polyphenols consumed were flavonoids, and among them the major subclass was flavanols. Alcoholic beverages were the major contributors to the total polyphenol intake (118.3 mg/d, SD: 127.5), and red wine contributed 17.7% of total polyphenols consumed. Polyphenol intake was highest among alcohol drinkers, high educational level, high income, and physical active people. Flavonoids were the highest ingested polyphenols. Alcoholic beverages were the major contributors to the total polyphenol intake, mainly red wine.

## Introduction

Unhealthy and stressful lifestyles may promote radical oxygen species formation [1]. Oxidative stress appears in the body when the radical formation exceeds the endogenous antioxidant mechanisms which leads to the appearance of diseases (i.e.: neurodegenerative diseases, cardiovascular diseases, cancer, atherosclerosis and diabetes) [2,3].

Polyphenols are found abundantly in our diets, and the increasing interest in these phytonutrients is due to their role in health. Polyphenols trap and scavenge free radicals, regulate nitric oxide, decrease leukocyte immobilization, induce apoptosis and exhibit phytoestrogenic activity according to in vitro studies [4,5]. This may contribute to their potential benefits on human health, including decreased risk of diabetes [6], certain cancers [7] and cardiovascular disease occurrence and mortality [8–10].

All polyphenols have at least one aromatic ring structure with one or more hydroxyl group [7]. They can be classified in several classes and subclasses according to the number of rings and the structural elements that bind them to one another [11]. The polyphenol content in the plants differs depending on the plant type. Cinnamic acid is found in all parts of fruits although it is mostly concentrated in the outer layer. Flavanones are found in tomatoes and aromatic plants. Isoflavones are found in leguminous plants mainly soybeans, and they have structural similarities to estrogens, which confer them pseudohormonal properties including the ability to bind to estrogen receptors. Flavanols exist as monomers and polymers and are found in fruits, red wine, green tea and chocolate. Finally, stillbenes are found in wines [11]. Nevertheless, polyphenols are affected by many factors as the ripeness at the time of harvest [11,12], environmental factors as the soil type, the exposure to sun and light, the rainfall, the green house agriculture, and are also affected by processing, storage and culinary preparation [11].

Great focus is on polyphenols and their benefits; however, data regarding their consumption at the population level is not enough to suggest optimal intake levels and set dietary recommendations [13]. Polyphenol intake has been assessed in Mediterranean area, including Spain [14], France [15], Sicily [16], but also in other countries as Poland [17], Denmark [18], Japan [19], and Brazil [20]. Some studies relied on the United States Department of Agriculture (USDA) database to estimate the polyphenol intake [21]; however, the Phenol Explorer database release 3.6 holds data on polyphenol content in 555 food items and introduces data on the effects of food processing on the polyphenol content [22] which provides more accuracy.

Oxidative stress accumulates with age and causes diseases [23]; hence, the aim of this study was to assess the polyphenol estimated intake and dietary sources among old adults from Mallorca Island (a Mediterranean region), who are more vulnerable to diseases caused by oxidative stress.

## Methods

### Study population

The sample consisted of 211 participants (53% women) engaged in a study conducted from 2013 to 2014 in Mallorca island. The study assesses the effect of lifestyle factors on the health of older adults living in Mallorca Island. Men aged between 55 and 80 and women aged between 60 and 80 were recruited in the study. Exclusion criteria included being institutionalized, suffering from a physical or mental illness which would have limited their participation in physical fitness or their ability to respond to questionnaires, chronic alcoholism or drug addiction and intake of drugs for clinical research over the past year.

Sociodemographic and lifestyle characteristics were collected from each participant. Educational level was ranked into primary school studies, secondary school studies and university graduate. The income was considered low if it was lower than 600 euros per month, medium if it was between 600 and 900 euros per month and high if it was higher than 900 euros per month. The participants were classified as well as smokers and nonsmokers, alcohol drinkers and nondrinkers, and physically active and inactive. Individuals with ≤1.5 hours/week of physical activity (n = 105) were categorised as “inactive” and they were recruited in social and municipal clubs, and health centres. Individuals who practice >1.5 hours/week of physical activity (n = 106) were categorised as “active” and they were recruited in social and municipal clubs, health centres and sport clubs. The study was conducted according to the guidelines laid down in the Declaration of Helsinki and all procedures were approved by the Balearic Islands Ethics Committee (approval reference number n° IB/2251/14 PI). Written informed consents were obtained from all participants.

### Anthropometric measurements

Anthropometric measurements were performed by well-trained dieticians who underwent identical and rigorous training as an effort to minimize the effects of inter-observer variation. Height was measured using an anthropometer (Kawe 44444, Asperg, Germany) with the subject’s head in the Frankfurt plane. Body weight was determined using a digital scale (Tefal, sc9210, Rumilly, France). The participants were weighed barefoot, noting and subtracting the weight of the clothes. Weight and height measures were used to calculate body mass index (BMI, kg/m^2^). According to the anthropometric reference parameters for the Spanish elderly [24,25], the prevalence of normal-weight, overweight, and obesity were defined as BMI ≤26.9 kg/m^2^, BMI 27.0–29.9 kg/m^2^, and BMI ≥30.0 kg/m^2^ respectively for those older than 65 and BMI ≤25.0kg/m^2^, BMI 25.0–29.0 kg/m^2^, and BMI ≥29.0 kg/m^2^ respectively for younger participants.

### Assessment of polyphenol content

Polyphenol content was assessed by two non-consecutive 24-h recalls. Licensed dieticians administered the recalls and verified and quantified the food records. Volumes and portion sizes were reported in natural units, household measures or with the aid of a manual of photographs [26]. The recall diets yielded 449 food items including food recipes. After excluding animal foods, commercial food products, deserts with high sugar content and dairy products that have no traces of polyphenols, food items were researched using polyphenol explorer (http://phenol-explorer.eu/). The polyphenol explorer provides the polyphenol content of food using data obtained by five analytical methods (chromatography, chromatography with hydrolysis, Folin assay, pH differential methods and normal phase HPLC) [27]. The very few food items that were not found in the polyphenol database were researched using the United States Department of Agriculture´s National nutrient database [21], and the polyphenol content of mustard was found in the literature [28] because it was not found elsewhere. Ingredients from different food recipes were taken separately and then summed to obtain the polyphenol content of the recipe. The total polyphenol intake was calculated as the sum of polyphenol classes. Energy intakes ranged between 1082–3428 kcal/day for men and 870–2701 kcal/day for women.

### Statistical analysis

Statistics were performed using statistical software SPSS for Windows version 21 (IBM, Chicago, USA). Data are shown as mean, standard deviation (SD), median and interquartile range (IQR). Total polyphenol intake (expressed per mg) and energy adjusted polyphenol intake (expressed as mg per 1000 kcal) were assessed in the total sample and according to sociodemographic and lifestyle factors. The polyphenol intake increases with a higher consumption of food; hence adjusting the polyphenol intake to the energy intake gives a clearer illustration of the polyphenol intake. Non-parametric tests (Mann-Whitney U test or Kruskal-Wallis test) were used because the sample is not normally distributed. Results were considered statistically significant if *p*-value <0.05.

## Results

### Polyphenol intake (Table 1)

Polyphenol contents were found in 245 food products. The mean and median daily intake of polyphenol were 332.7 mg/d (SD: 197.4) and 299.5 mg/d (IQR: 250.4) respectively; and 187.5 mg/d (SD: 100.5) and 172.9 mg/d (IQR: 140.3) respectively after adjusting the variable for energy intake. The polyphenol intake was also calculated according to different sociodemographic factors. Men have higher polyphenol intake than women (median 343.2 mg/d for men and 276.6 mg/d for women); however, women showed higher polyphenol intake (median 173.2 mg/d for women and 161.8 mg/d for men) after adjusting for energy intake. Differences in polyphenol intake were also found according to age, educational level, and income; however, differences remained significant only for age after adjusting for energy intake. Alcohol drinkers’ showed higher polyphenols intake than non-drinkers, and physically active people consumed higher than physically inactive people. No significant differences in polyphenol intake were found for BMI range, and between smokers and non-smokers.

**Table 1:** Total polyphenol intake and energy adjusted polyphenol intake according to sociodemographic and lifestyle characteristics (n = 211).

		Polyphenol intake			Energy adjusted Polyphenol intake		
	n	mean (SD)	median (IQR)	*p*-value	mean (SD)	median (IQR)	*p*-value
**Total Population**	211	332.7 (197.4)	299.5 (250.4)		187.5 (100.5)	172.9 (140.3)	
**Sex**							
Men	99	375.8 (217.6)	343.2 (317.8)	0.006	187.1 (103.1)	161.8 (155.9)	0.889
Women	112	294.7 (169.8)	276.6 (188.5)		187.8 (98.7)	173.2 (134.9)	
**Age Class**							
50–63	74	284.4 (159.4)	260.2 (196.9)	0.045	149.8 (75.6)	129.9 (98.3)	<0.001
64–67	67	364.3 (212.8)	347.6 (265.6)		205.5 (105.5)	189.4 (122.9)	
≥68	70	353.5 (211.0)	304.0 (211.0)		209.9 (108.2)	201.0 (159.6)	
**Educational Level**							
Primary School	95	284.3 (156.3)	258.4 (207.6)	0.014	168.8 (85.7)	163.2 (108.8)	0.085
Secondary School	75	365.9 (200.1)	334.1 (312.2)		198.6 (108.1)	184.3 (116.1)	
University Graduate	41	384.4 (250.5)	343.2 (294.2)		210.3 (112.2)	191.2 (142.1)	
**Income**							
Low (<600 euros/month)	60	272.48 (167.3)	242.6 (221.6)	0.006	179.9 (101.2)	163.93 (134.8)	0.464
Medium (600–900 euros/month)	23	305.15 (167.3)	307.0 (265.3)		169.2 (86.7)	158.92 (141.8)	
High (>900 euros/month)	128	332.7 (208.9)	332.2 (282.9)		194.3 (102.5)	178.19 (141.3)	
**BMI Range**							
Normal	82	350.1 (196.5)	317.7 (258.4)	0.518	202.7 (99.6)	190.2 (140.7)	0.100
Overweight	86	324.8 (204.8)	290.7 (239.3)		182.1 (101.5)	163.3 (131.7)	
Obese	43	315.4 (185.7)	299.5 (267.4)		169.3 (98.4)	150.5 (155.6)	
**Smoking Status**							
No	194	335.2 (188.3)	308.1 (248.8)	0.147	189.1 (98.0)	175.1 (149.3)	0.188
Yes	17	305.0 (287.8)	263.0 (162.0)		169.3 (128.4)	149.1 (121.5)	
**Alcohol Drinking**							
No	95	261.5 (162.7)	223.1 (209.4)	<0.001	160.6 (97.5)	137.0 (127.0)	<0.001
Yes	116	391.1 (204.7)	353.0 (279.0)		209.5 (98.0)	201.8 (130.7)	
**Physical Activity**							
Inactive	105	293.4 (190.7)	263.03 (195.2)	0.001	169.9 (98.3)	150.5 (117.9)	0.005
Active	106	371.7 (197.2)	348.02 (291.7)		204.9 (100.1)	100.1 (153.1)	

Abbreviations: SD, standard deviation; IQR, interquartile range; BMI, body mass index.

Statistical analysis using Mann-Whitney U test or Kruskal-Wallis test.

### Polyphenol intake from foods (Table 2)

The most ingested polyphenols were flavonoids (170.3 mg/d, SD: 144.4); among the flavonoids the major subclass was flavanols (46.0 mg/d, SD: 57.7). Among the phenolic acid class, cinnamic acid intake (82.2 mg/d, SD: 92.9) was higher than benzoic acid intake (17.5 mg/d, SD: 90.6).

**Table 2:** Distribution (mg/recall) of the polyphenol classes and subclasses from food sources.

	Food sources										
Polyphenol class	Total Foods	Oils and seeds	Nonalcoholic beverages	Alcoholic beverages	Cereals	Sweets	Fruits	Nuts	Legumes	Others^1^	Vegetables
Total Polyphenols	332.7 (237.9)	26.2 (46.5)	32.1 (71.8)	118.3 (127.5)	25.2 (39.9)	17.3 (37.8)	98.6 (126.3)	28.1 (178.3)	8.0 (10.6)	11.1 (31.0)	68.5 (99.0)
**Flavonoids**	170.3 (144.4)	0.7 (5.5)	24.1 (71.5)	86.4 (97.9)	0.1 (0.6)	15.1 (34.3)	71.2 (88.5)	0.1 (0.3)	7.2 (10.6)	7.6 (28.0)	16.3 (28.2)
Flavanols	46.0 (57.7)	0	2.1 (5.3)	49.5 (55.7)	0	12.5 (32.0)	9.3 (19.6)	0.1 (0.3)	2.5 (5.2)	0.03 (0.1)	1.0 (4.0)
Flavonols	22.7 (29.9)	0.2 (1.7)	0.1 (0.4)	7.2 (8.1)	0	1.0 (2.5)	4.7 (7.0)	<0.01	1.9 (5.7)	2.3 (18.0)	11.4 (20.6)
Flavanones	30.7 (50.6)	0	3.1 (14.8)	1.0 (1.0)	0	0.1 (0.4)	29.2 (50.4)	0	0	0.1 (0.5)	0.1 (0.1)
Flavones	10.7 (20.3)	0.2 (0.9)	0.5 (2.2)	0	0.14(0.6)	1.5 (2.1)	2.0 (5.0)	0	0.2 (0.3)	2.2 (3.9)	5.1 (19.5)
Anthocyanin	36.7 (61.9)	0.3 (2.8)	0	23.0 (26.7)	0	0	25.7 (60.1)	0	1.9 (6.9)	0	0
Dihydrochalcones	0.3 (1.8)	0	0	0	0	0	0.3 (1.9)	0	0	0	0
Isoflavonoids	19.3 (71.1)	0	18.4 (70.3)	0	0	0	0	0	0.6 (2.0)	0.7 (11.5)	0
**Phenolic Acids**	100.0 (130.0)	5.0 (17.0)	7.7 (7.4)	21.9 (20.0)	0.4 (2.1)	2.0 (3.5)	21.7 (57.8)	27.3 (178.4)	0.8 (1.0)	1.1 (2.3)	47.4 (74.5)
Benzoic Acid	17.5 (90.6)	0.5 (1.8)	3.0 (5.0)	9.5 (8.6)	0.1 (0.1)	0.01 (0.1)	1.4 (4.5)	19.3 (176.9)	0.1 (0.2)	0.1 (0.2)	2.6 (11.1)
Cinnamic Acid	82.2 (92.9)	4.5 (15.9)	4.7 (7.0)	12.1 (11.5)	0.4 (2.0)	2.0 (3.5)	20.3 (55.9)	8.0 (29.2)	0.7 (0.9)	1.0 (2.2)	44.8 (73.2)
**Alkylphenols**	25.3 (39.2)	0	0.02 (0.03)	<0.01	25.3 (39.6)	0.1 (0.2)	0	0	0	0	0
**Alkylmethoxyphenols**	0.1 (0.2)	0	0.02 (0.03)	0.1 (0.3)	0	<0.01	0	0	0	<0.0	0
**Lignans**	7.2 (15.6)	2.3 (14.4)	<0.01	0	0.04 (0.1)	<0.01	4.8 (7.1)	<0.01	0	0.1 (0.4)	0.4 (1.4)
**Stillbenes**	2.6 (4.4)	0	0	4.7 (5.1)	0	<0.01	0.1 (0.2)	<0.01	0.02 (0.03)	0	0
**Tyrosol**	20.6 (21.9)	17.5 (21.1)	0.04 (0.3)	4.5 (4.4)	0	0.0 (0.1)	0	0	0	1.9 (2.6)	0
**Other polyphenols**	2.4 (3.8)	0.5 (1.6)	0.2 (0.9)	1.0 (0.9)	0	0.0 (0.0)	0.8 (2.2)	0.7 (1.0)	0	0.3 (2.4)	<0.01

Values are expressed as mean (standard deviation).

^1^Others include appetizers, pastries and salted pastries, broth, chocolate, biscuits, sauces, condiments, precooked dishes, desert, jam.

Alcoholic beverages were the highest contributors (118.3 mg/d, SD: 127.5) to the total polyphenol intake; but fruits also were important contributors (98.6 mg/d, SD: 126.3). Food groups provided polyphenols differently. Flavonoids´ main sources were alcoholic beverages and fruits. The stilbene intake was mainly provided by alcoholic beverages and phenolic acids by vegetables. Tyrosol was provided mainly by oils and seeds, and alkylphenols mainly by cereals.

Different intake of polyphenol subclasses was also observed according to food groups: flavanols intake was mainly provided by alcoholic beverages; flavonols, flavones and cinnamic acid intake was mainly provided by vegetables. Flavanones and anthocyanin were highly obtained from fruits; and isoflavonoids mainly from nonalcoholic beverages. Benzoic acid was mostly found in nuts.

### Main food contributors of polyphenols (Table 3)

Red wine was the highest contributor of polyphenols intake (17.7%), as well as the highest contributor of flavonoids (26.8%) and most of their subclasses. The major contributors of flavanones, flavones, dihydrochalcones and isoflavonoids were orange, artichoke, peach juice and soy milk, respectively. Potatoes were the highest contributors of the phenolic acids. Further information is included in S1 Table.

**Table 3:** Main food contributors: Percentage of contribution to polyphenol classes and subclasses.

	1st	2nd	3rd
**Total Polyphenols**	red wine 17.7%	artichoke 6.2%	soy milk 5.4%
**Flavonoids**	red wine 26.8%	soy milk 10.8%	orange 9.5%
Flavanols	red wine 56.5%	pure chocolate 8.7%	plum 4.9%
Flavonols	red wine16.6%	spinach 13.0%	apple 11.9%
Flavanones	orange 52.6%	orange juice31.3%	commercial orange juice10.0%
Flavones	artichoke 43.8%	orange juice 13.7%	oli d´inca whole wheat cookies 5.2%
Anthocyanin	red wine 33.7%	black grapes 22.1%	cherry 17.5%
Dihydrochalcones	peach juice 80.0%	peach and grapes juice 10.3%	apple juice 9.7%
Isoflavonoids	soy milk 95.59%	soy yogurt 3.9%	soy sprouts 0.5%
**Phenolic Acids**	potato 16.5%	artichoke 15.5%	red wine 9.0%
Benzoic Acid	walnuts 23.5%	chestnuts19.4%	red wine 17.4%
Cinnamic Acid	potato 21.0%	artichoke 19.7%	red wine 6.7%
**Alkylphenols**	whole wheat bread 44.5%	rice cereals breakfast bars 19.2%	corn cereals breakfast bar 7.3%
**Alkylmethoxyphenols**	beer 74.4%	coffee beans 42.4%	powder coffee 1.8%
**Lignans**	melon 20.7%	mandarine 20.0%	orange 19.5%
**Stillbenes**	red wine 91.9%	white wine 3.8%	black grapes 1.5%
**Tyrosol**	virgin olive oil 31.4%	green olives 24.3%	olive oil 19.2%
**Other polyphenols**	walnuts 42.1%	orange juice 14.4%	green olives 11.7%

## Discussion

Several studies have used different methodologies to assess polyphenol intake in the past [14–20]; some of them were assessed in younger adults [15] and others in older adults [14,17,19,20]. However, to the best of our knowledge, this is the first study to assess the polyphenol intake in Spanish older adults using recall diets. In comparison with previous data, and despite the different methodologies used, the calculated mean polyphenol intake (332.8 mg/d) was relatively low compared to other studies in Sicily (663.7 mg/d) [16], Spain (820 mg/d) [14], France (1193 mg/d) [15], and Poland (1765 mg/d) [17], but higher than the intake estimated in Denmark (1–100 mg/d) [18], and similar to that observed in Brazil (377.5 mg/d) [20]. Alcohol drinkers in this study had higher intake than non-drinkers. Unlike the results obtained in Poland [17], no differences in polyphenol intake were registered between smokers and non-smokers.

Flavonoids were the main ingested polyphenol class in this study, similarly to previous Polish and Spanish studies [14,17] whereas phenolic acids were the main polyphenol class among French adults [15]. Moreover, phenolic acids and flavonoids were the main ingested polyphenols among Sicilian and Brazilian adults [16,23].

Nonalcoholic beverages were the main source of polyphenols among the French and Polish adults [15,17]; fruits and nuts among Spanish and Sicilian adults, respectively [14,16]; and coffee was the main contributor to the intake of other populations [14,15,19,20]. However, in the present study alcoholic beverages were the main source of polyphenols, being wine the highest food contributor. Flavonoid intake was mainly provided by red wine in this study as well; differently to a Polish study in which tea was the main source of flavonoids [17], and another Spanish study a Japanese study in which coffee and green tea were the main sources [19]. In another Spanish study on adult population [14], coffee was the main source of flavonoids; however, the present study has shown higher consumption of red wine than coffee consumption among the studied population (i.e. coffee mean intake was 35.8 mL/d, and red wine mean intake was 168.0 ml/d).

The polyphenol intake may differ according to the usual food intake in a region, but also according to the age, educational level, and lifestyle factors that impact on the eating habits; hence it is difficult to compare between populations. However, some results are coherent due to the fact that people with healthy lifestyle will have a healthy diet leading to high polyphenol intake (i.e. women tended to have as well higher awareness in nutrition than men) [29]. Adult population of Mallorca Island, the region where the study was conducted, showed different dietary pattern than other regions; hence, in the studied population red wine was the major source of polyphenols, differently from other populations that showed higher consumption of beverages containing caffeine (tea or coffee).

### Strengths and limitations

The present study has several important strengths. First, the polyphenol intake was estimated using recall diets instead of food frequency questionnaires that have been questioned in epidemiological studies [30,31]. Moreover, analyses were done per person (Table 1) and per recall (Table 2) to have a fair representation of the polyphenol intake in the sample without underestimating the difference of eating habits between people and the different dietary consumption in different days. Secondly, the Phenol Explorer database release 3.6 that introduces data on the effects of food processing on the polyphenol content was used [22]. The very few food items that were not found in the Polyphenol Explorer were researched using the USDA database to avoid the underestimation of their polyphenol contents.

This study has also several limitations that must be acknowledged. First, a limitation of the study was the relatively small sample size; for this reason, these findings cannot be generalized to the broader community based on this study alone. Second, food frequency questionnaires that have been questioned in epidemiological studies [32,33], but using two 24-h recall diets tend to underestimate the food intake over a large period. Third, despite the guidance of licensed dietitians, diet was self-reported. Moreover, conducting studies on polyphenols is faced with many limitations knowing that this phytonutrient is not very stable, and while the content of some products may be underestimated, the contents of others may be overestimated or misestimated.

Thus, the polyphenol contents were overestimated in 53 recipes, in which the polyphenol contents were calculated as the sum of the different ingredients in the recipe. Standard recipes were adopted; however, the effect of processing was not taken in consideration. Many of the recipes are typical from the region where the study was conducted (*coca de patata*, *coca de quarto*, *empanadas*, *turron de jijona*, *greixonera de brossat*, *galetes d´inca or whole wheat & olive oil cookies*…), however some of them are not (*mayonnaise*, *croissant*, *crepe*, *pizza*…). Moreover, the polyphenol contents of some commercial products may be underestimated due to the fact that estimating their polyphenol contents is not doable due to a lack of information on the exact recipes and ingredients (*yogurt with fruits*, *cookies*, *chips*, *Ketchup*…). An inaccurate estimation of polyphenols contents might have been done in 29 foods for they were not found so it was necessary to replace them by very similar products to estimate the polyphenol contents as for different brands of cereal bars, jams and fruit juices. Finally, 12 food products were not found in the Polyphenol Explorer, in the USDA database or the literature leading to an underestimation of their polyphenol contents (*algae*, *oat milk*, *linseed oil*, *nutmeg*…).

## Conclusions

Total polyphenol intake, the intake of different classes and subclasses of polyphenols, and their major food contributors have been assessed in adults living in a Mediterranean region as accurately as possible. Mean daily intake of polyphenol was calculated according to different sociodemographic factors including alcohol drinking, educational level, income, physical activity and age which were shown to be statistically significant. Flavonoids were the highest ingested polyphenols. Alcoholic beverages were the highest contributors of total polyphenol intake, mainly red wine. According to the importance of polyphenols in the diet and their probable benefits on health, further studies should be done to investigate the polyphenol intake, as well as the effect of food processing and the environmental factors on its content in the food. Furthermore, studies should investigate the polyphenol absorption by the body.

## Supporting information

S1 Table Food contributors table. (PDF)
