# Supplementary material for: Polyphenol estimated intake and dietary sources among older adults from Mallorca Island
Source: PLoS One. 2018 Jan 30;13(1):e0191573. doi: 10.1371/journal.pone.0191573 (PMC5790249; doi:10.1371/journal.pone.0191573)
Supplement: S1 Table — (PDF) [file pone.0191573.s001.pdf]

Alimento	Total_poly_adjusted	Favonoids_adjusted	phenolic_acid_adjusted	lignans_adjusted
ACEITE DE LINO	0	0	0	0
AGUA CON GAS EMBOTELLADA	0	0	0	0
AGUA CORRIENTE	0	0	0	0
AGUA SIN GAS EMBOTELLADA	0	0	0	0
ALA DE POLLO	0	0	0	0
ALBONDIGAS EN CONSERVA	0	0	0	0
ALGA KELP DESECADA	0	0	0	0
ALMEJA	0	0	0	0
ANCHOA EN ACEITE	0	0	0	0
ANGUILLA	0	0	0	0
ATUN AL NATURAL	0	0	0	0
ATUN EN ACEITE	0	0	0	0
AVENA PREPARADA CON AGUA	0	0	0	0
AZUCAR BLANCO	0	0	0	0
AZUCAR MORENO	0	0	0	0
BACALAO FRESCO	0	0	0	0
BACALOA SALADO	0	0	0	0
BACON	0	0	0	0
BARQUILLO	0	0	0	0
BARRA DE CHOCOLATE KIT-KAT	0	0	0	0
BARRITA DE CEREALES CON MELOCOTON Y ALB.	0	0	0	0
BEBIDA ISOTONICA	0	0	0	0
BOLLO DE LECHE	0	0	0	0
BOLLO RELLENO DE CHOCOLATE	0	0	0	0
BOLLO TIPO DONUT DE CHOCOLATE	0	0	0	0
BOQUERON	0	0	0	0
BUTIFARRA	0	0	0	0
CABALLA	0	0	0	0
CALAMAR	0	0	0	0
CALAMARES A LA ROMANA CONGELADOS	0	0	0	0
CALDO DE PESCADO	0	0	0	0
CALDO DE POLLO	0	0	0	0

CALDO EN CUBITOS DE CARNE	0	0	0	0
CALDO VEGETAL	0	0	0	0
CALLOS A LA MADRILEÑA	0	0	0	0
CANGREJO Y SIMILARES	0	0	0	0
CARACOL	0	0	0	0
CARAMELO	0	0	0	0
CARAMELOS DE GOMA (JELLYBEANS)	0	0	0	0
CARNE DE CERDO SEMIGRASA	0	0	0	0
CARNE DE VACA 2ªY3ª	0	0	0	0
CARNE DE VACA MAGRA	0	0	0	0
CARNE SEMIGRASA DE VACA	0	0	0	0
CAZON	0	0	0	0
CERDO MAGRO	0	0	0	0
CHORIZO	0	0	0	0
CHULETA DE CERDO	0	0	0	0
CHULETA DE CORDERO	0	0	0	0
CHURRO	0	0	0	0
CIGALA	0	0	0	0
CLARA DE HUEVO	0	0	0	0
COCOTIN (receta)	0	0	0	0
CODORNIZ	0	0	0	0
CONEJO	0	0	0	0
COSTILLA DE CERDO	0	0	0	0
CROQUETA DE JAMON CONGELADA	0	0	0	0
CROQUETA DE POLLO CONGELADAS	0	0	0	0
CUAJADA	0	0	0	0
DORADA	0	0	0	0
FLAN DE HUEVO	0	0	0	0
FLAN DE VAINILLA	0	0	0	0
FOIE GRAS	0	0	0	0
FRITOS DE MAIZ	0	0	0	0
FUET	0	0	0	0
GALLETA SALADA	0	0	0	0

GALLETA TIPO COOKIE	0	0	0	0
GALLETA TIPO DIGESTIVE	0	0	0	0
GALLETA TIPO DIGESTIVE CON CHOCOLATE	0	0	0	0
GALLINA ENTERA	0	0	0	0
GAMBA ROJA	0	0	0	0
GARBANZO	0	0	0	0
GARBANZO EN CONSERVA	0	0	0	0
GASEOSA	0	0	0	0
GELATINA	0	0	0	0
GINEBRA	0	0	0	0
GREIXONERA DE BROSSAT (receta)	0	0	0	0
GULAS SALTEADAS EN CONSERVA	0	0	0	0
HELADO CREMOSO	0	0	0	0
HELADO DE CHOCOLATE	0	0	0	0
HIGADO DE CERDO	0	0	0	0
HUEVO DE GALLINA	0	0	0	0
JALEA REAL	0	0	0	0
JAMON COCIDO	0	0	0	0
JAMON IBERICO	0	0	0	0
JAMON SERRANO	0	0	0	0
KEFIR	0	0	0	0
KETCHUP	0	0	0	0
LANGOSTINO	0	0	0	0
LECHE CONDENSADA AZUCARADA	0	0	0	0
LECHE DE CABRA	0	0	0	0
LECHE DE VACA DESNATADA	0	0	0	0
LECHE DE VACA ENTERA	0	0	0	0
LECHE DE VACA OMEGA 3	0	0	0	0
LECHE DE VACA SEMIDESNATADA	0	0	0	0
LECHE EN POLVO DESNATADA	0	0	0	0
LECHE EVAPORADA ENTERA	0	0	0	0
LECHE FERMENTADA CON LACTOBACILLUS CASI	0	0	0	0
LECHE FERMENTADA TIPO BIO CON FRUTAS	0	0	0	0

LECHE FERMENTADA TIPO BIO DESNATADO NA	0	0	0	0
LECHE FERMENTADA TIPO BIO NATURAL	0	0	0	0
LECITINA DE SOJA	0	0	0	0
LENGUADO	0	0	0	0
LOMO DE CERDO	0	0	0	0
LOMO EMBUCHADO	0	0	0	0
LONGANIZA	0	0	0	0
LUBINA	0	0	0	0
MAGDALENA	0	0	0	0
MAIZ EN GRANO HERVIDO EN LATA	0	0	0	0
MANITA DE CERDO	0	0	0	0
MANTECA DE CERDO	0	0	0	0
MANTEQUILLA	0	0	0	0
MARGARINA	0	0	0	0
MAYONESA LIGERA	0	0	0	0
MEJILLON	0	0	0	0
MEJILLON EN ESCABECHE	0	0	0	0
MERLUZA	0	0	0	0
MIEL	0	0	0	0
MORCILLA DE ARROZ	0	0	0	0
MORTADELA	0	0	0	0
MOUSSE DE CHOCOLATE	0	0	0	0
MUSLO DE POLLO	0	0	0	0
NATA LIQUIDA PARA COCINAR	0	0	0	0
NATA LIQUIDA PARA MONTAR	0	0	0	0
NATA MONTADA	0	0	0	0
NATILLAS	0	0	0	0
NATILLAS DE CHOCOLATE	0	0	0	0
NUEZ MOSCADA	0	0	0	0
PALITO DE CANGREJO	0	0	0	0
PALOMETA	0	0	0	0
PALOMITAS DE MAIZ	0	0	0	0
PANCETA	0	0	0	0

PASTA DE TE	0	0	0	0
PASTEL DE CHOCOLATE	0	0	0	0
PATATAS FRITAS DE BOLSA	0	0	0	0
PAVO	0	0	0	0
PECHUGA DE PAVO	0	0	0	0
PECHUGA DE PAVO EMBUTIDO	0	0	0	0
PECHUGA DE POLLO	0	0	0	0
PERCA	0	0	0	0
PESCADILLA	0	0	0	0
PIERNA DE CORDERO	0	0	0	0
POLLO ENTERO	0	0	0	0
PULMON DE CORDERO	0	0	0	0
PULPO	0	0	0	0
QUESO BRIE	0	0	0	0
QUESO CAMEMBERT	0	0	0	0
QUESO DE BURGOS	0	0	0	0
QUESO DE CABRA CURADO	0	0	0	0
QUESO DE CABRA SEMICURADO	0	0	0	0
QUESO DE CABRA TIERNO	0	0	0	0
QUESO EMMENTAL	0	0	0	0
QUESO EN LONCHAS	0	0	0	0
QUESO EN PORCIONES	0	0	0	0
QUESO EN PORCIONES DESCREMADO	0	0	0	0
QUESO FETA	0	0	0	0
QUESO GORGONZOLA	0	0	0	0
QUESO GOUDA	0	0	0	0
QUESO GRUYERE	0	0	0	0
QUESO HAVARTI	0	0	0	0
QUESO MANCHEGO CURADO	0	0	0	0
QUESO MANCHEGO SEMICURADO	0	0	0	0
QUESO MOZZARELLA	0	0	0	0
QUESO PARMESANO	0	0	0	0
QUESO ROQUEFORT	0	0	0	0

QUESO TIPO PETIT DE SABORES	0	0	0	0
RAPE	0	0	0	0
RAYA	0	0	0	0
REFRESCO SABOR COLA	0	0	0	0
REFRESCO SABOR COLA BAJO EN CALORIAS	0	0	0	0
REFRESCO SABOR COLA SIN CAFEINA	0	0	0	0
REFRESCO SABOR LIMON	0	0	0	0
REFRESCO SABOR NARANJA	0	0	0	0
REGALIZ	0	0	0	0
REQUESON	0	0	0	0
RODABALLO	0	0	0	0
ROLLITO DE PRIMAVERA CONGELADO	0	0	0	0
SACARINA	0	0	0	0
SAL COMUN	0	0	0	0
SAL HIPOSODICA	0	0	0	0
SALAMI	0	0	0	0
SALCHICHA FRESCA	0	0	0	0
SALCHICHA TIPO FRANKFURT	0	0	0	0
SALCHICHON	0	0	0	0
SALMON	0	0	0	0
SALMON AHUMADO	0	0	0	0
SALMONETE	0	0	0	0
SALSA AGRIDULCE	0	0	0	0
SALSA DE SOJA	0	0	0	0
SARDINA	0	0	0	0
SARDINA EN ACEITE	0	0	0	0
SEPIA	0	0	0	0
SOBRASADA	0	0	0	0
SOLOMILLO DE BUEY	0	0	0	0
SOLOMILLO DE CERDO	0	0	0	0
SOLOMILLO DE TERNERA	0	0	0	0
TERNERA DE 2ª Y 3ª	0	0	0	0
TERNERA MAGRA	0	0	0	0

TOCINO	0	0	0	0
TONICA	0	0	0	0
TRUCHA	0	0	0	0
YEMA DE HUEVO	0	0	0	0
YOGUR DESNATADO CON FRUTA	0	0	0	0
YOGUR DESNATADO DE SABORES	0	0	0	0
YOGUR DESNATADO NATURAL	0	0	0	0
YOGUR DESNATADO NATURAL AZUCARADO	0	0	0	0
YOGUR ENTERO CON FRUTA	0	0	0	0
YOGUR ENTERO DE SABORES	0	0	0	0
YOGUR ENTERO NATURAL AZUCARADO	0	0	0	0
YOGUR NATURAL ENTERO	0	0	0	0
YOGUR TIPO GRIEGO	0	0	0	0
CHAMPIÑON	1613,4	0	0	0
MOSTAZA	68,69	2,98	0	0
PAN BLANCO TOSTADO	0,1	0	0	0,1
PAN TIPO HAMBURGUESA	0,15	0	0	0,15
ALBARICOQUE SECO (OREJONES)	0,18	0	0	0,18
PAN DE MOLDE	0,29	0	0	0,29
BONIATO	0,47	0	0	0,47
PAN BLANCO TIPO BAGUETTE	5,83	0	0	5,83
PAN BLANCO SIN SAL	9,82	0	0	9,82
MELON	632,21	0	0	632,21
PASTA DE COLORES	0,97	0	0	0
PASTA INTEGRAL CRUDA	4,55	0	0	0
MUESLI	27,83	0	0	0
CEREALES EN POLVO SOLUBLES TIPO EKO	39,99	0	0	0
CEREALES DE DESAYUNO CON BASE DE ARROZ	57,13	0	0	0
CEREALES DE DESAYUNO CON BASE DE ARROZ,	85,7	0	0	0
CEREALES DE DESAYUNO CON BASE DE MAIZ	85,7	0	0	0
CEREALES DE DESAYUNO CON BASE DE TRIGO I	85,7	0	0	0
SALVADO DE TRIGO	142,83	0	0	0
PASTA	228,16	0	0	0

CEREALES DE DESAYUNO CON BASE DE TRIGO Y	257,09	0	0	0
CEREALES KELLOGG'S ALL-BRAN	599,89	0	0	0
CEREALES DE DESAYUNO CON BASE DE MAIZ AZ	771,28	0	0	0
CEREALES DE DESAYUNO CON BASE DE ARROZ Y	2042,47	0	0	0
SEMILLA DE SESAMO	465,89	0	0	465,89
BERRO	5,42	5,25	0	0,17
CAQUI	48,64	48,64	0	0
PUERRO	51,12	51,12	0	0
CANONIGOS	142,8	142,8	0	0
ENDIVIA	236,89	236,89	0	0
MANZANA	1137,48	1137,48	0	0
APIO	11,2	11,2	0	0
YOGUR DE SOJA	316,13	316,13	0	0
LECHE DE SOJA	7775,23	7775,23	0	0
ZUMO DE LIMÓN (crudo)	469,46	421,09	0	0
GUISANTE VERDE	0,24	0,24	0	0
GRANADA	7,43	7,43	0	0
MERMELADA DE FRESA	2,13	2,13	0	0
MERMELADA DE MORA	1,87	1,87	0	0
COÑAC	2,92	0	2,65	0
WHISKY	3,91	0	3,26	0
CREMA CATALANA (DIAL)	0,04	0	0,04	0
GALLETA INTEGRAL	130,18	71,01	0,22	0
BROTE DE SOJA	40,94	37,23	3,71	0
VINAGRE BALSAMICO	11,96	1,64	7,33	0
PAN DE CEBADA	70,54	0	5,5	0
BATIDO DE CACAO	169,84	162,64	7,2	0
DULCE DE MEMBRILLO Y PASTAS DE FRUTAS	11,54	3,5	8,03	0
MEMBRILLO	11,54	3,5	8,03	0
MERMELADA DE NARANJA	17,23	6,86	10,36	0
VINAGRE	90,96	12,48	55,78	0
ZUMO UVA Y MELOCOTON	101,8	61,1	40,7	0
MERMELADA DE ALBARICOQUE Y MELOCOTON	67,91	27,06	40,85	0

PAN DE CENTENO	708,75	0	55,28	0
ARROZ	87,65	0	87,65	0
ACEITE DE MAIZ	100,26	0	100,26	0
NUEZ SIN CASCARA	3035,26	0	2334,35	0
ZUMO DE MELOCOTON	788,95	473,53	315,43	0
PIPA DE GIRASOL PELADA SIN SAL	393,25	0	393,25	0
SEMILLA DE LINO	621,97	0	437,13	184,84
PIPA DE GIRASOL PELADA CON SAL	439,52	0	439,52	0
PEREJIL	0,14	0	0	0
CREMADILLO DE CREMA (receta)	6,35	0,49	0,43	0
TARTA MOUSSE DE LIMON (receta)	2,66	2,16	0,26	0
HUMMUS	4,02	0,02	0	0,63
ACEITE DE GIRASOL	0	0	0	0
TORTA DE ACEITE	5,9	0,02	0	0,51
CREPE (receta)	1,4	0,9	0,41	0
EMPANADILLA DE ATUN CONGELADA	7,38	0,03	0	0,63
SALSA BECHAMEL	2,57	1,66	0,74	0
TARTA DE ZANAHORIA (receta)	5,35	0,58	3,17	0,09
TOMILLO SECO	3,51	0	3,51	0
PUDIN (DIAL)	3,18	2,05	0,92	0
CREMA PASTELERA (receta)	3,58	2,31	1,04	0
SALSA AL CURRY	104,8	0,97	0	0,22
HARINA DE MAIZ	0,11	0	0,11	0
SALVADO DE AVENA	1,06	0	0	1,06
MANTECADO DE ALMENDRA (receta)	2,64	1,7	0,76	0,01
LECHE FRITA (receta)	6,16	2,62	1,18	0,18
TARTA DE PIÑA (receta)	0,35	0,18	0,08	0,07
ALBAHACA FRESCA	0	0	0	0
SALSA BOLOÑESA	1,74	0,23	1,49	0
ACEITUNA RELLENA DE PIMIENTO	27,72	0,04	9,41	0
PEPINILLOS EN VINARE EN CONSERVA	2,57	0,07	0	2,51
VINO DULCE (MALAGA, OPORTO)	5,34	1,7	2,7	0
GUINDILLA PICANTE	0,24	0,24	0	0

GALETES D'OLI O D'INCA INTEGRALS (receta)	462,37	233,89	0,73	2,9
TORTITA DE MAIZ	0,23	0	0,21	0
SOFRITO	2,68	0,53	1,58	0,05
TARTA REINA (receta)	1,39	1,07	0,26	0
CREMA DE CHOCOLATE	4,17	3,65	0,49	0
PIÑON SIN CASCARA	0,03	0	0	0,03
TARTA DE CREMA PASTELERA Y MANZANA	27,3	20,23	6,21	0
EMPANADA DE VERDURAS (RECETA)	16,08	8,15	3,56	0,31
VERMUT	5,16	1,19	3,09	0
ROBIOL CONFITURA (receta)	20,15	12,54	4,69	0,15
PIÑA EN ALMIBAR	0,29	0	0	0,29
TOMATE PELADO ENLATADO	4,16	0,29	3,87	0
TARTA DE QUESO (dial)	18,96	12,21	5,49	0
COCA DE PATATA (receta)	19,73	8,4	9,53	0,08
MASA DE HOJALDRE CONGELADA	20,26	13,05	5,87	0
AMARGO (RECETA)	0,06	0	0	0,06
COCARROI CEBOLLA Y SOBRASADA (receta)	12,23	8,21	1,6	0,18
ZUMO DE POMELO	53,17	47,08	0	0
AVENA	5,59	0	5,59	0
MAYONESA COMERCIAL	81,95	13,93	0	5,86
COCA DE QUARTO (receta)	39,63	33,1	4,36	0
ARROZ INTEGRAL	0,27	0	0,27	0
REPOLLO	0,09	0,03	0	0,06
GRELO	15,86	9,73	6,13	0
LIMON	43,04	43,01	0	0,02
ZUMO COMERCIAL SABOR FRUTAS TROPICALES	0	0	0	0
EMPANADA DE CARNE Y GUISANTES (receta)	37,28	18,2	8,16	0,78
ACEITE DE OLIVA VIRGEN EXTRA	87,39	2,14	0,43	1,51
ALIOLI	132,9	0,51	0	17,19
GALLETA DOBLE RELLENA DE CHOCOLATE	12,8	11,34	1,38	0
ZUMO DE TOMATE COMERCIAL	8,32	0,58	7,74	0
PEPINO	17,92	0,45	0	17,47
CAFÉ EN POLVO SOLUBLE	79,43	0	78,5	0

TURRON DE JIJONA	0,13	0	0	0,13
ACEITUNA RELLENA DE ANCHOA	239,12	0,39	81,16	0
NABO	0,32	0	0	0,32
RABANO	1,97	1,83	0	0,15
ESPARRAGO DE LATA	42,67	42,67	0	0
TARTA DE CHOCOLATE (DIAL)	21,48	19,15	2,25	0
EMPANADA DE CARNE (receta)	77,93	38,03	17,06	1,63
PAPAYA	0	0	0	0
ZUMO DE ZANAHORIA	60	0	60	0
ARANDANO	10,9	5,93	2,55	0
MERMELADA DE FRAMBUESA	6,6	4,61	2	0
COCA DE PIMIENTO ROJO (RECETA)	8,76	3,49	2,47	0,29
MANGO	2,32	2,32	0	0
ACEITUNA NEGRA SIN HUESO	950,63	266,93	231,24	0
JUDIA BLANCA	75,31	75,31	0	0
GERMEN DE TRIGO	50,07	0	50,06	0,01
HARINA DE TRIGO	92,27	59,44	26,72	0
ENSAIMADA	98,15	59,82	26,88	0,46
PIMIENTO DE PIQUILLO EN CONSERVA	0,89	0,17	0,73	0
BIZCOCHO	105,53	67,99	30,56	0
CACAHUETE SIN CASCARA	6,53	0	6,35	0
TOMATE TRITURADO	26,42	1,84	24,57	0
CROISANT	120,19	77,43	34,8	0
ALMENDRA SIN CASCARA	0,38	0	0	0,38
AJO	105,52	0	0	105,52
GALLETA CUBIERTA DE CHOCOLATE	49,09	41,88	6,52	0
MELOCOTON EN ALMIBAR	31,26	2,68	28,58	0
COCA DE VERDURA (RECETA)	277,66	227,56	27,21	1,29
ANIS	5,58	0	5,58	0
ZUMO DE MANZANA	174,73	136,88	37,88	0
COCA DE TREMPO (receta)	78,58	35,05	21,51	1,64
PIZZA MARGARITA CONGELADA	150,6	84,74	46,04	0,96
ZUMO DE PIÑA	0	0	0	0

GAZPACHO EN CONSERVA	29,44	8,77	7,2	4,2
LICOR DULCE	42,47	2,91	38,57	0
LECHE DE ALMENDRA	0,6	0	0	0,6
CAVA	151,72	7,67	83,16	0
DATIL	38,9	0	38,9	0
GALLETA TIPO MARIA	227,03	146,27	65,74	0
TOMATE FRITO EN CONSERVA	51,06	3,56	47,5	0
CACAO EN POLVO BAJO EN CALORIAS	54,91	51,16	3,71	0
BIZCOCHO DE CHOCOLATE	85,87	75,32	9,85	0
COL LOMBARDA	34,68	0,8	0	33,88
HIGO SECO	0,07	0	0	0,07
REMOLACHA	1,78	1,78	0	0,04
AGUACATE	2,13	2,13	0	0
CALABACIN	25,66	25,66	0	0
ESCAROLA	164,07	164,07	0	0
GALETES D'OLI O D'INCA (receta)	371,85	172,65	77,47	9,01
CANELA	2,06	0	2,06	0
CERVEZA SIN ALCOHOL	55,14	7,43	30,36	0
MORA	372,16	288,88	83,27	0
PAN DE MOLDE INTEGRAL	98,14	0	0	0
TRUFA DE CHOCOLATE (receta)	131,73	119,57	12,09	0
ACEITE DE OLIVA	1852,88	7,53	0	158,67
ACEITE DE OLIVA VIRGEN	2929,06	11,67	26,9	142,6
INFUSION DE MANZANILLA	97,81	97,81	0	0
CAFÉ DESCAFEINADO EN POLVO SOLUBLE	1241,31	0	1231,1	0
COL	1,62	0,54	0	1,08
HIGO	4,66	0	0	4,66
AVELLANA SIN CASCARA	10,83	10,83	0	0
ESPARRAGO VERDE	120,08	120,08	0	0
VINO BLANCO	427,63	135,74	216,22	0
CIRUELA SECA	223,87	2,88	221	0
JUDIA BLANCA EN CONSERVA	5,06	5,06	0	0
COLIFLOR	123,54	0	123,54	0

VINO ROSADO	178,67	41,25	106,93	0
COCARROI DE VERDURA (RECETA)	953,95	819,91	97,51	2,34
BOMBON	36,34	36,34	0	0
ACEITUNA VERDE SIN HUESO	3511,59	5,67	1191,84	0
ZUMO COMERCIAL DE NARANJA	1608,74	1532,15	0	0
JUDIA PINTA EN CONSERVA	65,99	65,99	0	0
HABA SECA	82,99	82,99	0	0
BRECOL	403,35	247,42	155,93	0
BERENJENA	53,58	0	53,58	0
CAFÉ EN GRANO	1844,1	0	1822,45	0
NISPERO	1771,11	0	1771,11	0
PAN INTTEGRAL TOSTADO	286,5	0	0	0
JUDIA PINTA	97,87	97,87	0	0
SANDIA	96,98	92,46	0	4,52
CALABAZA	40,91	38,32	0	2,59
JUDIA VERDE	648,82	648,82	0	0
ESPINACA	1247,21	1247,21	0	0
CEREZA	4336,37	2948,25	1388,12	0
PIÑA	19,48	0	0	19,48
CEBOLLA	427,98	427,98	0	0
PASA	48,53	1,81	46,72	0
NECTARINA	396,83	34,02	362,81	0
CASTAÑA	1822,91	0,08	1822,83	0
PIMIENTO VERDE	107,88	97,59	10,28	0
ZANAHORIA	1442,71	0	1442,71	0
ALCAPARRA	838,03	838,03	0	0
CHOCOLATE CON LECHE	106,34	106,34	0	0
CACAO EN POLVO AZUCARADO	466,76	434,88	31,5	0
LENTEJA EN CONSERVA	13,49	9,36	4,02	0
HABA FRESCA	234,41	234,41	0	0
MELOCOTON	486,52	41,71	444,82	0
ALBARICOQUE	694,37	298,04	396,33	0
ZUMO DE NARANJA	5024,38	4785,18	0	0

CERVEZA	716,38	146,4	499,71	0
TIRAMISU (DIAL)	587,42	530,76	54,15	0
PERA	1012,31	292,53	716,75	0
INFUSION GENERICA	607,55	607,55	0	0
PIMIENTO ROJO	24,43	4,52	19,91	0
CIRUELA	3876,06	2063,49	1812,57	0
KIWI	32,9	32,9	0	0
UVA NEGRA	4324,3	4228,08	79,63	0
FRESA Y FRESON	3521,92	3049,37	459,91	0
LECHUGA	1443,32	767,27	676,05	0
TOMATE	1188,08	82,82	1105,26	0
ACELGA	1843,51	189,12	1514,68	0,17
CHOCOLATE PURO	2086,77	1894,13	191,52	0
MANDARINA	611,07	0	0	611,07
PATATA NUEVA	7293,09	0	7293,09	0
PLATANO	312,25	189,8	122,45	0
ALCACHOFA	8816,84	1959,82	6857,01	0
PAN INTEGRAL	4729,31	0	0	0
NARANJA	7429,16	6831,61	0	597,56
VINO TINTO	25234,98	19209,14	3993,17	0
LENTEJA	179,17	124,39	53,23	0
Suma	142886,86	71687,2	44232,42	3058,12
	5,199330435	9,529748686	0	19,54010961
	17,6608122	26,79577386	9,027699592	0
	0,125392916	0,173517727	0,120341596	0

Stillbenes_adjusted	tyrosol_adjusted	alkylphenol_adjusted	alkylmethoxyphenol_adjusted	others_adjusted	flavonols_adjusted
0	0	0	0	0	0
0	0	0	0	0	0
0	0	0	0	0	0
0	0	0	0	0	0
0	0	0	0	0	0
0	0	0	0	0	0
0	0	0	0	0	0
0	0	0	0	0	0
0	0	0	0	0	0
0	0	0	0	0	0
0	0	0	0	0	0
0	0	0	0	0	0
0	0	0	0	0	0
0	0	0	0	0	0
0	0	0	0	0	0
0	0	0	0	0	0
0	0	0	0	0	0
0	0	0	0	0	0
0	0	0	0	0	0
0	0	0	0	0	0
0	0	0	0	0	0
0	0	0	0	0	0
0	0	0	0	0	0
0	0	0	0	0	0
0	0	0	0	0	0
0	0	0	0	0	0
0	0	0	0	0	0
0	0	0	0	0	0
0	0	0	0	0	0
0	0	0	0	0	0
0	0	0	0	0	0
0	0	0	0	0	0

0	0	0	0	0	0
0	0	0	0	0	0
0	0	0	0	0	0
0	0	0	0	0	0
0	0	0	0	0	0
0	0	0	0	0	0
0	0	0	0	0	0
0	0	0	0	0	0
0	0	0	0	0	0
0	0	0	0	0	0
0	0	0	0	0	0
0	0	0	0	0	0
0	0	0	0	0	0
0	0	0	0	0	0
0	0	0	0	0	0
0	0	0	0	0	0
0	0	0	0	0	0
0	0	0	0	0	0
0	0	0	0	0	0
0	0	0	0	0	0
0	0	0	0	0	0
0	0	0	0	0	0
0	0	0	0	0	0
0	0	0	0	0	0
0	0	0	0	0	0
0	0	0	0	0	0
0	0	0	0	0	0
0	0	0	0	0	0
0	0	0	0	0	0
0	0	0	0	0	0
0	0	0	0	0	0
0	0	0	0	0	0
0	0	0	0	0	0

0	0	0	0	0	0
0	0	0	0	0	0
0	0	0	0	0	0
0	0	0	0	0	0
0	0	0	0	0	0
0	0	0	0	0	0
0	0	0	0	0	0
0	0	0	0	0	0
0	0	0	0	0	0
0	0	0	0	0	0
0	0	0	0	0	0
0	0	0	0	0	0
0	0	0	0	0	0
0	0	0	0	0	0
0	0	0	0	0	0
0	0	0	0	0	0
0	0	0	0	0	0
0	0	0	0	0	0
0	0	0	0	0	0
0	0	0	0	0	0
0	0	0	0	0	0
0	0	0	0	0	0
0	0	0	0	0	0
0	0	0	0	0	0
0	0	0	0	0	0
0	0	0	0	0	0
0	0	0	0	0	0
0	0	0	0	0	0
0	0	0	0	0	0
0	0	0	0	0	0
0	0	0	0	0	0
0	0	0	0	0	0
0	0	0	0	0	0

0	0	0	0	0	0
0	0	0	0	0	0
0	0	0	0	0	0
0	0	0	0	0	0
0	0	0	0	0	0
0	0	0	0	0	0
0	0	0	0	0	0
0	0	0	0	0	0
0	0	0	0	0	0
0	0	0	0	0	0
0	0	0	0	0	0
0	0	0	0	0	0
0	0	0	0	0	0
0	0	0	0	0	0
0	0	0	0	0	0
0	0	0	0	0	0
0	0	0	0	0	0
0	0	0	0	0	0
0	0	0	0	0	0
0	0	0	0	0	0
0	0	0	0	0	0
0	0	0	0	0	0
0	0	0	0	0	0
0	0	0	0	0	0
0	0	0	0	0	0
0	0	0	0	0	0
0	0	0	0	0	0
0	0	0	0	0	0
0	0	0	0	0	0
0	0	0	0	0	0
0	0	0	0	0	0
0	0	0	0	0	0
0	0	0	0	0	0

0	0	0	0	0	0
0	0	0	0	0	0
0	0	0	0	0	0
0	0	0	0	0	0
0	0	0	0	0	0
0	0	0	0	0	0
0	0	0	0	0	0
0	0	0	0	0	0
0	0	0	0	0	0
0	0	0	0	0	0
0	0	0	0	0	0
0	0	0	0	0	0
0	0	0	0	0	0
0	0	0	0	0	0
0	0	0	0	0	0
0	0	0	0	0	0
0	0	0	0	0	0
0	0	0	0	0	0
0	0	0	0	0	0
0	0	0	0	0	0
0	0	0	0	0	0
0	0	0	0	0	0
0	0	0	0	0	0
0	0	0	0	0	0
0	0	0	0	0	0
0	0	0	0	0	0
0	0	0	0	0	0
0	0	0	0	0	0
0	0	0	0	0	0
0	0	0	0	0	0
0	0	0	0	0	0
0	0	0	0	0	0
0	0	0	0	0	0

0	0	0	0	0	0
0	0	0	0	0	0
0	0	0	0	0	0
0	0	0	0	0	0
0	0	0	0	0	0
0	0	0	0	0	0
0	0	0	0	0	0
0	0	0	0	0	0
0	0	0	0	0	0
0	0	0	0	0	0
0	0	0	0	0	0
0	0	0	0	0	0
0	0	0	0	0	0
0	0	0	0	0	0
0	0	0	0	0	0
0	0	0	0	0	0
0	0	0	0	0	0
0	0	0	0	0	0
0	0	0	0	0	0
0	0	0	0	0	0
0	0	0	0	0	0
0	0	0	0	0	0
0	0	0	0	0	0
0	0	0	0	0	0
0	0	0	0	0	0
0	0	0	0	0	0
0	0	0	0	0	0
0	0	0	0	0	0
0	0	0	0	0	0
0	0	0	0	0	0
0	0	0	0	0	0
0	0	0	0	0	0
0	0	0	0	0	0

0	0	0	0	0	0
0	0	0	0	0	0
0	0	0	0	0	0
0	0	0	0	0	0
0	0	0	0	0	0
0	0	0	0	0	0
0	0	0	0	0	0
0	0	0	0	0	0
0	0	0	0	0	0
0	0	0	0	0	0
0	0	0	0	0	0
0	0	0	0	0	0
0	0	0	0	0	0
0	0	0	0	0	0
0	0	0	0	0	0
0	0	0	0	0	0
0	0	0	0	0	0
0	0	0	0	0	0
0	0	0	0	0	0
0	0	0	0	0	0
0	0	0	0	0	0
0	0	0	0	0	0
0	0	0	0	0	0
0	0	0,97	0	0	0
0	0	4,55	0	0	0
0	0	27,83	0	0	0
0	0	39,99	0	0	0
0	0	57,13	0	0	0
0	0	85,7	0	0	0
0	0	85,7	0	0	0
0	0	85,7	0	0	0
0	0	142,83	0	0	0
0	0	228,16	0	0	0

0	0	257,09	0	0	0
0	0	599,89	0	0	0
0	0	771,28	0	0	0
0	0	2042,47	0	0	0
0	0	0	0,01	0	0
0	0	0	0	0	5,25
0	0	0	0	0	48,64
0	0	0	0	0	51,12
0	0	0	0	0	142,8
0	0	0	0	0	236,89
0	0	0	0	0	1137,48
0	0	0	0	0	0
0	0	0	0	0	0
0	0	0	0	0	0
0	0	0	0	48,38	0
0	0	0	0	0	0
0	0	0	0	0	0
0	0	0	0	0	0
0	0	0	0	0	0
0	0	0	0	0,27	0
0	0	0	0	0,66	0
0	0	0	0	0	0
0	0	58,95	0	0	0
0	0	0	0	0	0
0	2,6	0	0	0,38	0,1
0	0	65,04	0	0	0
0	0	0	0	0	0
0	0	0	0	0	0,35
0	0	0	0	0	0,35
0	0	0	0	0	4,33
0	19,8	0	0	2,9	0,73
0	0	0	0	0	0
0	0	0	0	0	17,08

0	0	653,47	0	0	0
0	0	0	0	0	0
0	0	0	0	0	0
0	0	0	0	700,91	0
0	0	0	0	0	0
0	0	0	0	0	0
0	0	0	0	0	0
0	0	0	0	0	0
0	0	0	0	0,14	0
0	0	0,05	0	0	0
0	0	0,06	0	0,18	0
0	3,35	0	0	0,03	0
0	0	0	0	0	0
0	5,33	0	0	0,04	0
0	0	0,09	0	0	0
0	6,67	0	0	0,05	0
0	0	0,17	0	0	0
0	0,94	0,06	0	0,5	0
0	0	0	0	0	0
0	0	0,21	0	0	0
0	0	0,24	0	0	0
0	2,37	0	0	101,24	0,96
0	0	0	0	0,01	0
0	0	0	0	0	0
0	0	0,17	0	0	0
0	1,9	0,27	0	0,01	0
0	0	0,02	0	0	0
0	0	0	0	0	0
0,02	0,01	0	0	0,01	0,14
0	16,74	0	0	1,53	0
0	0	0	0	0	0,07
0,53	0,21	0	0	0,21	0,24
0	0	0	0	0	0,15

0	30,56	194,07	0	0,23	0
0	0	0	0	0,02	0
0	0,51	0	0	0	0,48
0	0	0,06	0	0	0
0	0	0,04	0	0	0,35
0	0	0	0	0	0
0	0	0,85	0	0	11,34
0	3,29	0,75	0	0,02	0,85
0,33	0,56	0	0	0	0
0	1,58	1,07	0	0,12	0,05
0	0	0	0	0	0
0	0	0	0	0	0,15
0	0	1,25	0	0	0
0	0,85	0,86	0	0,01	0
0	0	1,34	0	0	0
0	0	0	0	0	0
0	1,86	0,37	0	0,01	4,65
0	0	0	0	6,09	0,63
0	0	0	0	0	0
0	61,7	0	0	0,46	0,14
0	0	1	0	1,17	0,57
0	0	0	0	0	0
0	0	0	0	0	0,03
0	0	0	0	0	9,73
0	0	0	0	0	0,43
0	0	0	0	0	0
0	8,22	1,86	0	0,06	0
0	83,3	0	0	0	0
0	114,34	0	0	0,86	0
0,01	0	0,07	0	0	1,13
0	0	0	0	0	0,3
0	0	0	0	0	0,45
0	0	0,1	0,41	0,43	0

0	0	0	0	0	0
0	144,36	0	0	13,21	0
0	0	0	0	0	0
0	0	0	0	0	1,83
0	0	0	0	0	42,67
0,01	0	0,06	0	0	1,95
0	17,19	3,9	0	0,13	0
0	0	0	0	0	0
0	0	0	0	0	0
2,42	0	0	0	0	0,64
0	0	0	0	0	2,84
0	2,14	0,34	0,01	0,03	0,08
0	0	0	0	0	0
0	443,79	0	0	8,67	82,55
0	0	0	0	0	74,38
0	0	0	0	0	0
0	0	6,11	0	0	0
0	4,82	6,14	0	0,04	0
0	0	0	0	0	0,11
0	0	6,98	0	0	0
0,18	0	0	0	0	0
0	0	0	0	0	0,95
0	0	7,95	0	0	0
0	0	0	0	0	0
0	0	0	0	0	0
0,02	0	0,67	0	0	3,72
0	0	0	0	0	0
0	13,78	2,21	0,06	5,69	17,76
0	0	0	0	0	0
0	0	0	0	0	4,8
0	17,44	2,79	0,08	0,25	5,95
0	10,16	8,64	0	0,08	0,32
0	0	0	0	0	0

0	9,14	0	0	0,13	4,32
0	0	0	0	0,99	0
0	0	0	0	0	0
0,25	60,64	0	0	0,74	0,25
0	0	0	0	0	0
0	0	15,02	0	0	0
0	0	0	0	0	1,84
0	0	0	0	0,04	0
0,04	0	0,66	0	0	7,26
0	0	0	0	0	0,54
0	0	0	0	0	0
0	0	0	0	0	0,46
0	0	0	0	0	2,13
0	0	0	0	0	25,66
0	0	0	0	0	208,71
0	94,54	17,71	0	0,63	0
0	0	0	0	0	0
0	17,35	0	0	0	0
0	0	0	0	0	18,52
0	0	98,14	0	0	0
0,07	0	0	0	0	12,59
0	1674,12	0	0	12,55	0
0	2733,69	0	0	0	0
0	0	0	0	0	0
0	0	8,53	0	0	0
0	0	0	0	0	0,54
0	0	0	0	0	0
0	0	0	0	0	0
0	0	0	0	0	617,58
42,44	16,82	0	0	16,42	18,82
0	0	0	0	0	2,88
0	0	0	0	0	4,99
0	0	0	0	0	0

11,26	19,24	0	0	0	0
0	24,7	7,87	0	1,62	52,46
0	0	0	0	0	0
0	2120,03	0	0	194,05	0
0	0	0	0	76,59	37,26
0	0	0	0	0	14,34
0	0	0	0	0	0
0	0	0	0	0	247,42
0	0	0	0	0	0
0	0	2,23	9,45	9,96	0
0	0	0	0	0	0
0	0	286,5	0	0	0
0	0	0	0	0	21,29
0	0	0	0	0	0
0	0	0	0	0	0
0	0	0	0	0	452,13
0	0	0	0	0	1247,21
0	0	0	0	0	0
0	0	0	0	0	0
0	0	0	0	0	427,98
0	0	0	0	0	1,81
0	0	0	0	0	0
0	0	0	0	0	0,08
0	0	0	0	0	49,37
0	0	0	0	0	0
0	0	0	0	0	838,03
0	0	0	0	0	0
0	0	0,02	0	0,37	0
0,12	0	0	0	0	1,4
0	0	0	0	0	0
0	0	0	0	0	0
0	0	0	0	0	37,9
0	0	0	0	239,21	116,37

0	62,46	1,95	29,28	46,85	17,57
0	0	2	0,04	0,48	0
0	0	0	0	3,03	46,73
0	0	0	0	0	0
0	0	0	0	0	3,02
0	0	0	0	0	136,75
0	0	0	0	0	0
16,59	0	0	0	0	145,99
12,64	0	0	0	0	83,75
0	0	0	0	0	695,73
0	0	0	0	0	42,84
0	0	0	0	0	189,12
1,12	0	0	0	0	199,5
0	0	0	0	0	0
0	0	0	0	0	0
0	0	0	0	0	0
0	0	0	0	0	0
0	0	4729,31	0	0	0
0	0	0	0	0	15,21
1015,17	852,19	0	0	165,31	1594,94
1,55	0	0	0	0	18,78
1104,77	8705,29	10627,51	39,34	1664	9580,58
0	0	0	0	0	0,158758656
91,8897146	9,789334991	0	0	9,934495192	16,64763511
0,140300696	0	0	0	0	0,196021535

flavone_adjusted	isoflavone_adjusted	flavanone_adjusted	flavanols_adjusted	anthocyanin_adjusted	dihydrochalcones_adjusted
0	0	0	0	0	0
0	0	0	0	0	0
0	0	0	0	0	0
0	0	0	0	0	0
0	0	0	0	0	0
0	0	0	0	0	0
0	0	0	0	0	0
0	0	0	0	0	0
0	0	0	0	0	0
0	0	0	0	0	0
0	0	0	0	0	0
0	0	0	0	0	0
0	0	0	0	0	0
0	0	0	0	0	0
0	0	0	0	0	0
0	0	0	0	0	0
0	0	0	0	0	0
0	0	0	0	0	0
0	0	0	0	0	0
0	0	0	0	0	0
0	0	0	0	0	0
0	0	0	0	0	0
0	0	0	0	0	0
0	0	0	0	0	0
0	0	0	0	0	0
0	0	0	0	0	0
0	0	0	0	0	0
0	0	0	0	0	0
0	0	0	0	0	0
0	0	0	0	0	0
0	0	0	0	0	0
0	0	0	0	0	0

0	0	0	0	0	0
0	0	0	0	0	0
0	0	0	0	0	0
0	0	0	0	0	0
0	0	0	0	0	0
0	0	0	0	0	0
0	0	0	0	0	0
0	0	0	0	0	0
0	0	0	0	0	0
0	0	0	0	0	0
0	0	0	0	0	0
0	0	0	0	0	0
0	0	0	0	0	0
0	0	0	0	0	0
0	0	0	0	0	0
0	0	0	0	0	0
0	0	0	0	0	0
0	0	0	0	0	0
0	0	0	0	0	0
0	0	0	0	0	0
0	0	0	0	0	0
0	0	0	0	0	0
0	0	0	0	0	0
0	0	0	0	0	0
0	0	0	0	0	0
0	0	0	0	0	0
0	0	0	0	0	0
0	0	0	0	0	0
0	0	0	0	0	0
0	0	0	0	0	0
0	0	0	0	0	0
0	0	0	0	0	0
0	0	0	0	0	0

0	0	0	0	0	0
0	0	0	0	0	0
0	0	0	0	0	0
0	0	0	0	0	0
0	0	0	0	0	0
0	0	0	0	0	0
0	0	0	0	0	0
0	0	0	0	0	0
0	0	0	0	0	0
0	0	0	0	0	0
0	0	0	0	0	0
0	0	0	0	0	0
0	0	0	0	0	0
0	0	0	0	0	0
0	0	0	0	0	0
0	0	0	0	0	0
0	0	0	0	0	0
0	0	0	0	0	0
0	0	0	0	0	0
0	0	0	0	0	0
0	0	0	0	0	0
0	0	0	0	0	0
0	0	0	0	0	0
0	0	0	0	0	0
0	0	0	0	0	0
0	0	0	0	0	0
0	0	0	0	0	0
0	0	0	0	0	0
0	0	0	0	0	0
0	0	0	0	0	0
0	0	0	0	0	0
0	0	0	0	0	0
0	0	0	0	0	0

0	0	0	0	0	0
0	0	0	0	0	0
0	0	0	0	0	0
0	0	0	0	0	0
0	0	0	0	0	0
0	0	0	0	0	0
0	0	0	0	0	0
0	0	0	0	0	0
0	0	0	0	0	0
0	0	0	0	0	0
0	0	0	0	0	0
0	0	0	0	0	0
0	0	0	0	0	0
0	0	0	0	0	0
0	0	0	0	0	0
0	0	0	0	0	0
0	0	0	0	0	0
0	0	0	0	0	0
0	0	0	0	0	0
0	0	0	0	0	0
0	0	0	0	0	0
0	0	0	0	0	0
0	0	0	0	0	0
0	0	0	0	0	0
0	0	0	0	0	0
0	0	0	0	0	0
0	0	0	0	0	0
0	0	0	0	0	0
0	0	0	0	0	0
0	0	0	0	0	0
0	0	0	0	0	0
0	0	0	0	0	0
0	0	0	0	0	0

0	0	0	0	0	0
0	0	0	0	0	0
0	0	0	0	0	0
0	0	0	0	0	0
0	0	0	0	0	0
0	0	0	0	0	0
0	0	0	0	0	0
0	0	0	0	0	0
0	0	0	0	0	0
0	0	0	0	0	0
0	0	0	0	0	0
0	0	0	0	0	0
0	0	0	0	0	0
0	0	0	0	0	0
0	0	0	0	0	0
0	0	0	0	0	0
0	0	0	0	0	0
0	0	0	0	0	0
0	0	0	0	0	0
0	0	0	0	0	0
0	0	0	0	0	0
0	0	0	0	0	0
0	0	0	0	0	0
0	0	0	0	0	0
0	0	0	0	0	0
0	0	0	0	0	0
0	0	0	0	0	0
0	0	0	0	0	0
0	0	0	0	0	0
0	0	0	0	0	0
0	0	0	0	0	0
0	0	0	0	0	0
0	0	0	0	0	0

0	0	0	0	0	0
0	0	0	0	0	0
0	0	0	0	0	0
0	0	0	0	0	0
0	0	0	0	0	0
0	0	0	0	0	0
0	0	0	0	0	0
0	0	0	0	0	0
0	0	0	0	0	0
0	0	0	0	0	0
0	0	0	0	0	0
0	0	0	0	0	0
0	0	0	0	0	0
0	0	0	0	0	0
0	0	0	0	0	0
0	0	0	0	0	0
0	0	0	0	0	0
0	0	0	0	0	0
0	0	0	0	0	0
0	0	0	0	0	0
0	0	0	0	0	0
0	0	0	0	0	0
0	0	0	0	0	0
0	0	0	0	0	0
0	0	0	0	0	0
0	0	0	0	0	0
0	0	0	0	0	0
0	0	0	0	0	0
0	0	0	0	0	0
0	0	0	0	0	0
0	0	0	0	0	0
0	0	0	0	0	0
0	0	0	0	0	0

0	0	0	0	0	0
0	0	0	0	0	0
0	0	0	0	0	0
0	0	0	0	0	0
0	0	0	0	0	0
0	0	0	0	0	0
0	0	0	0	0	0
0	0	0	0	0	0
0	0	0	0	0	0
0	0	0	0	0	0
0	0	0	0	0	0
0	0	0	0	0	0
0	0	0	0	0	0
0	0	0	0	0	0
0	0	0	0	0	0
0	0	0	0	0	0
0	0	0	0	0	0
0	0	0	0	0	0
0	0	0	0	0	0
0	0	0	0	0	0
0	0	0	0	0	0
0	0	0	0	0	0
0	0	0	0	0	0
0	0	0	0	0	0
0	0	0	0	0	0
0	0	0	0	0	0
0	0	0	0	0	0
0	0	0	0	0	0
0	0	0	0	0	0
0	0	0	0	0	0
0	0	0	0	0	0
0	0	0	0	0	0
0	0	0	0	0	0

0	0	0	0	0	0
0	0	0	0	0	0
0	0	0	0	0	0
0	0	0	0	0	0
0	0	0	0	0	0
0	0	0	0	0	0
0	0	0	0	0	0
0	0	0	0	0	0
0	0	0	0	0	0
0	0	0	0	0	0
0	0	0	0	0	0
11,2	0	0	0	0	0
0	316,13	0	0	0	0
0	7775,23	0	0	0	0
53,66	0	367,42	0	0	0
0	0	0	0,24	0	0
0	0	0	7,43	0	0
0	0	0	0,9	1,23	0
0	0	0	0	1,87	0
0	0	0	0	0	0
0	0	0	0	0	0
0	0	0	0	0	0
71,01	0	0	0	0	0
0	37,23	0	0	0	0
0	0	0	1,55	0	0
0	0	0	0	0	0
0	0	0	162,64	0	0
0	0	0	3,15	0	0
0	0	0	3,15	0	0
0	0	0	2,53	0	0
0	0	0	11,75	0	0
0	0	0	49,5	0	11,7
0	0	0	9,98	0	0

0	0	0	0	0	0
0	0	0	0	0	0
0	0	0	0	0	0
0	0	0	0	0	0
0	0	0	383,63	0	90,68
0	0	0	0	0	0
0	0	0	0	0	0
0	0	0	0	0	0
0	0	0	0	0	0
0,49	0	0	0	0	0
0,79	0	1,37	0	0	0
0,02	0	0	0	0	0
0	0	0	0	0	0
0,02	0	0	0	0	0
0,9	0	0	0	0	0
0,03	0	0	0	0	0
1,66	0	0	0	0	0
0,58	0	0	0	0	0
0	0	0	0	0	0
2,05	0	0	0	0	0
2,31	0	0	0	0	0
0,01	0	0	0	0	0
0	0	0	0	0	0
0	0	0	0	0	0
1,7	0	0	0	0	0
2,62	0	0	0	0	0
0,18	0	0	0	0	0
0	0	0	0	0	0
0	0	0,04	0,04	0	0
0,04	0	0	0	0	0
0	0	0	0	0	0
0	0	0,12	1,04	0,02	0
0,09	0	0	0	0	0

233,89	0	0	0	0	0
0	0	0	0	0	0
0	0	0,06	0	0	0
0,57	0	0	0,51	0	0
0,34	0	0	2,96	0	0
0	0	0	0	0	0
8,28	0	0	0,61	0	0
7,27	0	0,01	0,01	0	0
0	0	0	1	0	0
10,71	0	1,78	0	0	0
0	0	0	0	0	0
0	0	0,14	0	0	0
12,21	0	0	0	0	0
8,4	0	0	0	0	0
13,05	0	0	0	0	0
0	0	0	0	0	0
3,57	0	0	0	0	0
0	0	46,45	0	0	0
0	0	0	0	0	0
0,75	0	13,05	0	0	0
12,7	0	19,83	0	0	0
0	0	0	0	0	0
0	0	0	0	0	0
0	0	0	0	0	0
1,48	0	41,1	0	0	0
0	0	0	0	0	0
18,18	0	0	0,02	0	0
2,14	0	0	0	0	0
0,51	0	0	0	0	0
0,66	0	0	9,56	0	0
0	0	0,28	0	0	0
0	0	0	0	0	0
0	0	0	0	0	0

0	0	0	0	0	0
0,39	0	0	0	0	0
0	0	0	0	0	0
0	0	0	0	0	0
0	0	0	0	0	0
0,6	0	0	16,6	0	0
38,03	0	0	0	0	0
0	0	0	0	0	0
0	0	0	0	0	0
0	0	0	5,29	0	0
0	0	0	0	1,77	0
3,37	0	0	0,04	0	0
0	0	0	2,32	0	0
45,82	0	0	0	138,56	0
0	0,74	0	0,19	0	0
0	0	0	0	0	0
59,44	0	0	0	0	0
59,82	0	0	0	0	0
0,06	0	0	0	0	0
67,99	0	0	0	0	0
0	0	0	0	0	0
0	0	0,89	0	0	0
77,43	0	0	0	0	0
0	0	0	0	0	0
0	0	0	0	0	0
6,57	0	0	31,59	0	0
0	0	0	2,68	0	0
21,5	0	0,22	0,25	0	0
0	0	0	0	0	0
0	0	0	121,13	0	11
28,48	0	0,3	0,31	0	0
84,12	0	0,3	0	0	0
0	0	0	0	0	0

3,99	0	0,18	0,28	0	0
0	0	0	2,91	0	0
0	0	0	0	0	0
0	0	0	7,43	0	0
0	0	0	0	0	0
146,27	0	0	0	0	0
0	0	1,72	0	0	0
0	0	0	51,16	0	0
6,41	0	0	61,65	0	0
0,26	0	0	0	0	0
0	0	0	0	0	0
1,31	0	0	0	0	0
0	0	0	0	0	0
0	0	0	0	0	0
0	0	0	0	0	0
172,65	0	0	0	0	0
0	0	0	0	0	0
0	0	0,62	6,81	0	0
0	0	0	20,11	250,26	0
0	0	0	0	0	0
0	0	0	106,97	0	0
7,53	0	0	0	0	0
11,67	0	0	0	0	0
0	0	0	97,81	0	0
0	0	0	0	0	0
0	0	0	0	0	0
0	0	0	0	0	0
0	0	0	10,83	0	0
0	0	0	0	0	0
0	0	9,21	83,28	1,6	0
0	0	0	0	0	0
0	0,05	0	0,01	0	0
0	0	0	0	0	0

0	0	0	34,66	0	0
80,47	0	24,39	0	0	0
0	0	0	36,34	0	0
5,67	0	0	0	0	0
196,65	0	1298,24	0	0	0
0	1,68	0	0	49,93	0
0	0	0	82,99	0	0
0	0	0	0	0	0
0	0	0	0	0	0
0	0	0	0	0	0
0	0	0	0	0	0
0	0	0	0	0	0
0	2,53	0	0	74,05	0
92,46	0	0	0	0	0
38,32	0	0	0	0	0
0	0	0	196,68	0	0
0	0	0	0	0	0
0	0	0	238,26	2710,15	0
0	0	0	0	0	0
0	0	0	0	0	0
0	0	0	0	0	0
0	0	0	34,02	0	0
0	0	0	0	0	0
48,22	0	0	0	0	0
0	0	0	0	0	0
0	0	0	0	0	0
0	0	0	106,34	0	0
0	0	0	434,88	0	0
1,21	0	0	6,71	0	0
0	0	0	234,41	0	0
0	0	0	41,71	0	0
0	0	0	260,14	0	0
614,18	0	4054,63	0	0	0

0	0	9,76	117,12	0	0
19,14	0	0	511,62	0	0
0	0	0	245,79	0	0
0	0	0	607,55	0	0
1,51	0	0	0	0	0
0	0	0	954,42	972,32	0
0	0	0	32,9	0	0
0	0	0	665,02	3417,54	0
0	0	0	329,95	2635,3	0
71,54	0	0	0	0	0
0	0	39,98	0	0	0
0	0	0	0	0	0
0	0	0	1694,63	0	0
0	0	0	0	0	0
0	0	0	0	0	0
0	0	0	189,8	0	0
1959,82	0	0	0	0	0
0	0	0	0	0	0
0	0	6816,4	0	0	0
0	0	200,24	10945,71	5201,6	0
16,37	0	0	89,24	0	0
4473,34	8133,59	12948,73	19372,74	15456,2	113,38
0	0	52,64145596	0	0	0
0	0	1,546406482	56,50057762	33,65380883	0
0,365945803	0	0	0,460647281	0	0

benzoic_acid_adjusted	cinamic_acid_adjusted	folin_essay_adjusted
0	0	0
0	0	0
0	0	0
0	0	0
0	0	0
0	0	0
0	0	0
0	0	0
0	0	0
0	0	0
0	0	0
0	0	0
0	0	0
0	0	0
0	0	0
0	0	0
0	0	0
0	0	0
0	0	0
0	0	0
0	0	0
0	0	0
0	0	0
0	0	0
0	0	0
0	0	0
0	0	0
0	0	0
0	0	0
0	0	0
0	0	0
0	0	0

0	0	0
0	0	0
0	0	0
0	0	0
0	0	0
0	0	0
0	0	0
0	0	0
0	0	0
0	0	0
0	0	0
0	0	0
0	0	0
0	0	0
0	0	0
0	0	0
0	0	0
0	0	0
0	0	0
0	0	0
0	0	0
0	0	0
0	0	0
0	0	0
0	0	0
0	0	0
0	0	0
0	0	0
0	0	0
0	0	0
0	0	0
0	0	0
0	0	0

0	0	0
0	0	0
0	0	0
0	0	0
0	0	0
0	0	0
0	0	0
0	0	0
0	0	0
0	0	0
0	0	0
0	0	0
0	0	0
0	0	0
0	0	0
0	0	0
0	0	0
0	0	0
0	0	0
0	0	0
0	0	0
0	0	0
0	0	0
0	0	0
0	0	0
0	0	0
0	0	0
0	0	0
0	0	0
0	0	0
0	0	0
0	0	0
0	0	0

0	0	0
0	0	0
0	0	0
0	0	0
0	0	0
0	0	0
0	0	0
0	0	0
0	0	0
0	0	0
0	0	0
0	0	0
0	0	0
0	0	0
0	0	0
0	0	0
0	0	0
0	0	0
0	0	0
0	0	0
0	0	0
0	0	0
0	0	0
0	0	0
0	0	0
0	0	0
0	0	0
0	0	0
0	0	0
0	0	0
0	0	0
0	0	0
0	0	0

0	0	0
0	0	0
0	0	0
0	0	0
0	0	0
0	0	0
0	0	0
0	0	0
0	0	0
0	0	0
0	0	0
0	0	0
0	0	0
0	0	0
0	0	0
0	0	0
0	0	0
0	0	0
0	0	0
0	0	0
0	0	0
0	0	0
0	0	0
0	0	0
0	0	0
0	0	0
0	0	0
0	0	0
0	0	0
0	0	0
0	0	0
0	0	0
0	0	0

0	0	0
0	0	0
0	0	0
0	0	0
0	0	0
0	0	0
0	0	0
0	0	0
0	0	0
0	0	0
0	0	0
0	0	0
0	0	0
0	0	0
0	0	0
0	0	0
0	0	0
0	0	0
0	0	0
0	0	0
0	0	0
0	0	0
0	0	0
0	0	0
0	0	0
0	0	0
0	0	0
0	0	0
0	0	0
0	0	0
0	0	0
0	0	0
0	0	0

0	0	0
0	0	0
0	0	0
0	0	0
0	0	0
0	0	0
0	0	0
0	0	0
0	0	0
0	0	0
0	0	0
0	0	0
0	0	0
0	0	0
0	0	0
0	0	0
0	0	0
0	0	0
0	0	0
0	0	0
0	0	0
0	0	0
0	0	0
0	0	0
0	0	0
0	0	0
0	0	0
0	0	0
0	0	0
0	0	0
0	0	0
0	0	0
0	0	0

0	0	0
0	0	0
0	0	0
0	0	0
0	0	0
0	0	0
0	0	0
0	0	0
0	0	0
0	0	0
0	0	0
0	0	0
0	0	0
0	0	0
0	0	0
0	0	0
0	0	0
0	0	0
0	0	0
2,65	0	0
3,26	0	0
0	0,04	0
0	0,22	0
2,15	1,56	0
3,62	3,72	0
0,6	4,9	0
0	7,2	0
0	8,03	0
0	8,03	0
0	10,36	0
27,53	28,25	0
0	40,7	0
0	40,85	0

6,01	49,27	0
31,65	56	0
0	100,26	0
2206,6	127,74	0
0	315,43	0
0	393,25	0
0	437,13	0
0	439,52	0
0	0	0,89
0	0,22	1,82
0	0,26	2,18
0	0	2,86
0	0	2,88
0	0	3,17
0	0,4	3,35
0	0	3,96
0,01	0,74	6,15
1,57	1,6	6,35
0,07	3,44	7,26
0,01	0,91	7,62
0,01	1,03	8,57
0	0	9,48
0,03	0,08	10,2
0	1,06	10,56
0,01	0,76	10,59
0,01	1,16	10,85
0	0,08	11,35
0	0	12,05
0,05	1,44	12,85
1,11	8,3	12,93
0	0	13,04
1,24	1,42	16,05
0	0	16,32

0	0,73	18,15
0,06	0,15	20,4
0	1,58	22,18
0,01	0,25	24,64
0	0,49	27,18
0	0	27,87
0,04	6,17	30,78
0,04	3,53	39,51
0,78	2,31	41,11
0,05	4,64	42,02
0	0	44,37
0	3,87	45,06
0,06	5,43	45,38
0,04	9,49	45,96
0,06	5,8	48,5
0	0	49,5
0,02	1,58	53,41
0	0	53,6
0	5,59	55,44
0	0	58,78
0,05	4,31	61,79
0,15	0,11	63,33
0	0	66,47
0	6,13	69,49
0	0	69,73
0	0	71,6
0,09	8,07	72,3
0,13	0,22	77,2
0	0	85,81
0	1,37	86,16
0	7,74	90,12
0	0	90,77
0	78,5	98,68

0	0	101,87
9,54	71,61	111,53
0	0	115,81
0	0	128,5
0	0	138,24
0,11	2,14	148,76
0,19	16,87	151,2
0	0	158,4
0,15	59,85	173,46
0	2,55	175,77
2	0	186,07
0,32	2,15	191,29
0	0	195,44
70,26	160,99	195,67
0	0	205,11
1,1	48,97	208,87
0,29	26,43	220,85
0,29	26,58	225,03
0	0,73	251,94
0,33	30,23	252,59
0	6,35	254,29
0	24,57	286,13
0,38	34,42	287,68
0	0	294,1
0	0	297,72
0,03	6,49	301,06
0	28,58	320,94
0,85	26,33	323,25
1,98	3,6	324
3,6	34,3	356,45
1,07	20,4	360,15
0,41	45,63	414,45
0	0	415,28

0,65	6,54	419,08
37,52	1,05	445,14
0	0	463,88
2,23	80,93	465,3
16,36	22,54	475,23
0,72	65,02	543,41
0	47,5	553,07
0,01	3,7	562,42
0,03	9,82	563,78
0	0	563,79
0	0	576
0	0	582,56
0	0	589,47
0	0	590,23
0	0	676,17
0,79	76,52	695,96
0,2	1,86	727,5
19,2	11,15	755,79
72,75	10,54	826,4
0	0	856,53
0	12,09	936,88
0	0	994,23
0	5,07	1048,92
0	0	1077,3
1231,1	1,68	1185,4
0	0	1194,2
0	0	1206,2
0	0	1276,42
0	0	1284,87
99,3	113,31	1285,28
0	221	1374,25
0	0	1377,3
38,83	84,54	1404,46

26,86	79,89	1424,7
7,88	89,6	1555,37
0	0	1614,7
140,14	1051,6	1637,9
0	0	1686,36
0	0	1692,07
0	0	1746,51
0	155,93	1767,1
29,13	24,45	2197,28
0	1822,45	2290,95
97,63	1673,7	2431,04
0	0	2500,54
0	0	2508,91
0	0	2584,86
0	0	2585,63
0	0	2590,36
0	0	2594,8
0	1388,12	2765,17
0	0	3032,16
0	0	3599,46
1,14	45,58	3780,75
0	362,81	4074,57
1822,83	0	4135,01
0	10,28	4148,64
3,61	1439,1	4170,87
0	0	4608
0	0	4724,5
0,05	31,45	4780,6
0,59	3,39	4806,48
0	0	4933,03
0	444,82	4995,53
0	396,33	5250,18
0	0	5266,82

353,31	132,74	5432,42
0,15	53,99	5705,99
32,77	683,98	6549,06
0	0	6691,8
0	19,91	6908,08
0	1812,57	8339,23
0	0	8446,37
0	79,63	8767,58
204,69	255,23	10440,12
0	676,05	11789,79
0	1105,26	12868,96
995,02	519,66	14141,54
0	191,52	14841,84
0	0	14851,2
0	7293,09	18066,81
122,45	0	18943,02
0	6857,01	38735,36
0	0	41276,21
0	0	42359,61
1629,87	2326,05	50171,93
7,92	45,31	63697,08
9378,4	34779,55	550854,79
0	0	7,689796071
17,37897722	6,687981874	9,108013747
0,084449373	0,130277706	11,56331599
